# Adoptive transfer of rapidly-generated multivirus-specific T cells to treat Adv, EBV, CMV, BK and HHV6 infections of HSCT recipients

**DOI:** 10.1186/2051-1426-1-S1-O2

**Published:** 2013-11-07

**Authors:** Anastasia Papadopoulou, Usha Katari, Ulrike Gerdemann, Caridad Martinez, Kathy Leung, George Carrum, Adrian Gee, Juan Vera, Robert Krance, Malcolm Brenner, Cliona Rooney, Helen Heslop, Ann Leen

**Affiliations:** 1 Baylor College of Medicine, Texas Children's Hospital, The Methodist Hospital, Houston, TX, USA

## 

Severe and fatal viral infections remain common after HSCT. Adoptive transfer of cytotoxic T lymphocytes (CTLs) specific for EBV, CMV and Adv antigens can treat infections that are impervious to conventional therapies, but extension to additional viruses has been limited by competition between virus-derived antigens and laborious manufacturing procedures. We are now evaluating whether infusion of rapidly-generated donor-derived pentavalent T cell lines (pCTL), stimulated just once with peptide libraries spanning immunogenic EBV (LMP2, EBNA1, BZLF1), Adv (Hexon, Penton), CMV (pp65, IE1), BK (Large T, VP1) and HHV6 (U90, U11, U14) antigens, and expanded in the presence of IL4+7 in a G-Rex device, is safe and effective in HSCT recipients with active infections. With NHLBI-PACT support, 35 clinical-grade pCTL lines have been generated. From 30x10^6^ PBMCs we prepared a mean of 374x10^6^ cells (range 99-713x10^6^) over 9-11 days. The lines were polyclonal, comprising both CD4+ (57±5%) and CD8+ (35±5%) cells, with specificity for CMV (IE1: 337±141; pp65: 1059±479 SFC/2x10^5^), EBV (LMP2: 175±87; EBNA1: 116±44; BZLF1: 129±88), Adv (Hexon: 446±153; Penton: 317±108), BK (Large T: 130±67; VP1: 231±104) and HHV6 (U90: 66±50; U11: 36±18; U14: 82±21) and no alloreactivity against recipient PHA blasts (mean Cr_51_ release 1% 20:1 E:T). To date we have infused 10 patients on study; 4 on DL1 (5x10^6^/m2), 4 on DL2 (1x10^7^/m2) and 2 on DL3 (2x10^7^/m2), with no adverse events. Three patients were infused prophylactically (38-43 days post-HSCT), while 7 received the cells as treatment for one or more active infections at 59-139 days post-HSCT. Based on viral load measurements by day 42, the pCTLs were successful in controlling active CMV (1 complete (CR) and 1 partial response (PR)), EBV (2 CRs, including a case of frank PTLD); Adv (1 CR); BK (1 CR, 3 PR) and HHV6 (1 CR) infections. Additionally, 1 patient received cells off study as treatment for widespread rituximab-resistant EBV-PTLD. Post-infusion there was an immediate decline in her EBV viral load with PTLD resolution, coincident with an increase in circulating EBV-specific T cells. However, the profound anti-tumor activity mediated by the rapidly-expanding EBV-directed T cells also produced a transient systemic inflammatory response syndrome in this patient, which was controlled with steroids and anti-TNFR antibody, with no long term adverse effects. In summary, infusion of pCTLs has thus far proven safe and is associated with the appearance of virus-reactive T cells in peripheral blood and subsequent virus clearance.

